# Rhabdomyosarcoma of the Middle Ear Case Report

**DOI:** 10.3390/children11121496

**Published:** 2024-12-08

**Authors:** Stoyan Stefanov Markov, Mariya Ivanova Spasova, Neofit Iuriev Spasov, Petya Petkova Markova

**Affiliations:** 1 Department of Otorhinolaryngology, Medical University of Plovdiv, 4000 Plovdiv, Bulgaria; 2 Department of Otorhinolaryngology, University Hospital “St. George”, 4000 Plovdiv, Bulgaria; 3 Department of Pediatrics, Medical University of Plovdiv, 4000 Plovdiv, Bulgaria; mariya.spasova@mu-plovdiv.bg (M.I.S.); neofit.spasov@mu-plovdiv.bg (N.I.S.); petya.markova@mu-plovdiv.bg (P.P.M.); 4 Depatrment of Pediatrics, University Hospital “St. George”, 4000 Plovdiv, Bulgaria

**Keywords:** rabdomyosarkoma, malignant soft tissue tumors, temporal bone tumors

## Abstract

Background: Rhabdomyosarcoma (RMS) is a highly malignant soft tissue tumor derived from primitive embryonal mesenchymal tissue that differentiates into striated skeletal muscle. Despite the improved outcome based on the EFS and OS using the three different treatment modalities-chemotherapy, radiotherapy and surgical treatment, the survival of patients depends on their IRS groups—pathological and surgical. On the other hand in the last thirty years a great improvement of the five-year overall survival (OS) of children with RMS have been observed based on the results of large multinational collaborative trials and successive studies dedicated to children, though prognosis is variable and dependent on several factors including histologic variant, primary sites of the tumor, extent of disease (disease resectability), and molecular-level characteristics. Case presentation: We present a clinical case of a five-year-old male with initial complains of left side peripheral facial nerve palsy and secondary cervical and retroauricular lymphadenomegaly. After an exam, surgery of the temporal bone, CT and MRI embryonal type of rhabdomyosarcoma was diagnosed, and adjuvant chemotherapy was initiated in combination with concomitant local radiotherapy. Results: The results show that in these areas surgery itself is insufficient for RMS treatment(usually it is limited to taking a biopsy only). The combination of chemotherapy and local control with radiotherapy achieved a good result in our patient. Conclusions: Middle ear Embryonal Rhabdomyosarcoma is a common solid tumor, which could mimic middle ear inflammation or mastoid inflammation in patients. The multimodal approach seemed to be the ideal management of RMS. It involves a combination of chemotherapy and local control with surgery and/or radiotherapy.

## 1. Introduction

Rhabdomyosarcoma (RMS) is the most common soft tissue head and neck sarcoma in children, accounting 3–4% of all childhood malignancies [1,2] as the most common primary site for RMS is in the head and neck region (35–40% of cases) [3].

The actual etiology of rhabdomyosarcoma as most of the tumors in the pediatric age is unknown. Most RMS cases result from sporadic mutations, whereas there are cases that are associated with Li–Fraumeni syndrome, Beckwith–Wiedemann syndrome, Noonan syndrome or cardiofaciocutaneous syndrome [3,4]. Embryonal RMS have characteristically been linked to loss of heterozygosity at a specific site on the short arm of chromosome 11 (11p15). Alveolar RMS has translocations involving the FKHR gene on the long arm of chromosome 13 with genes of the PAX family on either chromosome 2 (PAX3) or chromosome 1 (PAX7). TP53 mutations can be found in both embryonal and alveolar histologic RMS subtypes. There are also data for levated n-myc expression (10% of patients with alveolar), as well as point mutations in N-ras and K-ras oncogenes (usually embryonal RMS) [5]. It is the most common soft tissue tumor in children [6].

Four different histological subentities of RMS have been discribed: embryonal, alveolar, anaplastic, and mixed-type/sclerotic RMS [1,3,7]. The most common in children (60%) is the embryonal subtype [3,4]. The RMS of the head and neck can be further classified according to their localisation as orbital, parameningeal, or non-parameningeal/non-orbital [1]. Parameningeal localization includes nasal cavities, paranasal sinuses, nasopharynx, skull base as well as infratemporal and pterygopalatine fossa, the middle ear and mastoid process [3]. Due to their localization parameningeal RMS are likely to infiltrate central nervous tissue. They show a tendency to grow unnoticed and are often asymptomatic in early tumor stages [7]. Non-orbital/Non-parameningeal RMS tumors can grow up from both superficial locations (external ear, scalp, cheek), or deep locations (parotid, oropharynx, palate, neck and larynx) [5,8]. Orbital lesions involve orbita and soft tissues in and round it—eyeball, eyelid, ophthalmic nerve, vessels, and extraocular muscles.

Pathologically RMS is one of the small, round blue cell tumors in childhood. Along the myogenesis the RMS cells (myoblasts) can have variable differentiation. Histological pictures of embryonal RMS show both spindle and primitive round cells that can be tightly packed or loosely dispersed upon a myoid background. Rhabdomyoblasts which are more highly differentiated cells, can assume a variety of shapes (tadpole, racquet, strap cells), and reveal cross-striations [5].

In around 80% of cases in children younger than 5 years of age, and 64% in patients between 15–19 years embryonal RMS is diagnosed. According to the literature, the main primary sites in the head are orbit (25%) and epipharynx (25%), followed by paranasal cavities, ears, mouth, neck and parotid region with approximately 10%. RMS of the orbit has the best prognosis and parameningeal has the worst. The rate of distant metastasis at the time of diagnosis is between 9% and 23% [8,9,10]. RMS first presentation depends on the lesion location mostly.

### 1.1. Parameningeal RMS

The primary ear localisation of the RMS presents with bloody otorrhea, hearing loss, otalgia and central or peripheral facial nerve palsy. Nose and throat involvement suggests tumor-associated rhinosinusitis with symptoms of nasal obstruction and mucopurulent, bloody or mixed rhinorrhea. If the tumor invades the orbit—orbital symptoms may also occur. Nasopharyngeal RMS complains usually are sleep apnea, upper airway obstruction and abducens nerve palsy. The intracranial extension of parameningeal RMS can cause oculomotor, trochlear, abducens nerve involvement and can cause diplopia.

### 1.2. Orbital RMS

The most typical symptoms of that localization are rapid unilateral eyeball proptosis and inferior or inferiotemporal displacement of the globe. Ophthalmoplegia, blepharoptosis, ocular or orbital pain can also present.

### 1.3. Non-Orbital/Non-Parameningeal RMS

These tumors ca cause dysphagia, dysphonia, and respiratory airway obstruction syndrome. If parotid gland is involved facial nerve paralysis may also present. The superficial non-parameningeal RMS can only present as a painless tumour mass.

*Physical exam* is initial for diagnosis. Tumour mass in front or under the ear, lesion extending through the tympanic membrane and appears to be arising from the ear canal. Tumor mass in the nasal cavity or soft tissue lesion in any one of the three paranasal sinuses (maxillary, ethmoid, and sphenoid), pharyngeal narrowing caused by a tumor mass at the nasopharynx or oropharynx eyeball dislocation and tumour lesion involving the orbit are most widespread initial exam findings.

### 1.4. Imaging Techniques

Magnetic resonance imaging (MRI)T is the best imaging tool for RMS of the head and neck. It has excellent spatial resolution, superior soft-tissue contrast, multiplanar imaging, opportunity to evaluate the disease activity after contrast enhancement and with the lack\presence of restriction of the water molecules and absence of ionizing radiation [5].

The most widespread imaging test for primary site visualization and verification the diagnosis is CT scan. On it RMS appears as a heterogeneous mass, often with focal necrosis. CT images are very helpful for bone erosion identification [11].

Helpful tool for stagging the malignant tumor process is Positron Emission Tomography (PET). It is even more important for distant metastases and lymph node metastases detection [12]. It is used for morphologic and metabolic response evaluation in course of the multimodal treatment.

### 1.5. Biopsy

Taking a biopsy is the essential part of the diagnostic process. An open biopsy under general anesthesia is preferred. If not possible, fine needle biopsy is a matter of choice. For primary RMS tumours in the sinonasal and nasopharyngeal cavities an endoscopic biopsy is indicated.

If frozen section is obtained at the time of open biopsy and it is suggestive of RMS, bone marrow aspiration for cytogenetic analysis can be performed under the same anaesthesia [5].

### 1.6. Staging the RMS Tumors

There are few classifications of RMS. Staging based on the site and size of the tumor and the presence or absence of metastases (TNM) is shown on Table 1.

Statistical results show that despite all modern therapies the survival of patients is still mostly dependent on their IRS surgical and pathological groups [14] (Table 2). Survival is highest in group I and lowest in group IV, with embryonal disease doing better than alveolar rhabdomyosarcoma [15,16].

### 1.7. Treatment

**Figure 1:**
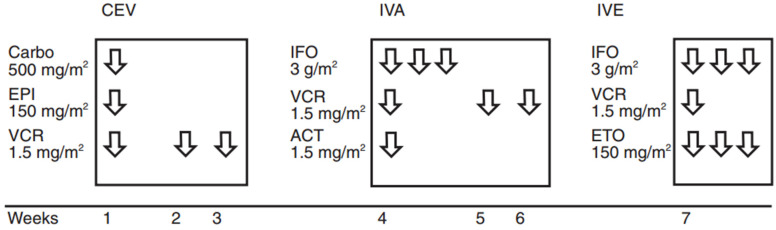
CWS 2009 protocols, CEVAIE regimen. Carbo = carboplatin; EPI = epirubicin; VCR = vincristine; IFO = ifosfamide (associated with Mesna); ACT = actinomycin; D; ETO = etoposide.

Taking into consideration that these are complicated cases, a multidisciplinary approach is required. The RMS therapy includes chemotherapy, surgery and radiation as standard treatment. Chemotherapy is based on the CWS protocols (Figure 1) and EuRhab indications. There are different treatment schemes, according to the stage, primary site, pathological subtype, metastases and effect from surgery (R_0_, R_1_).

Surgical treatment especially for the parameningeal primary tumors is challenging—complete resection often is impossible or can lead to major deterioration of the ECOG or Lansky\Karnofsky status. Non-parameningeal RMS are more amenable to complete surgical excision. Complete surgical resection with negative margins offers the best chance of survival [17].

Radiotherapy is an essential part of all treatment protocols, with the exception of cases with fully resected tumor. It can replace surgery as local control in R_1_ or non-resectable cases or as adjuvant treatment in R_0_ tumours, which are high risk as primary sites. It is often used for metastatic sites control if they are not responding to systemic chemotherapy both metabolically and morphologically.

## 2. Case Report

We present a clinical case of 5-year-old child, admitted in a pediatric department due to left-sided peripheral facial nerve palsy. No B symptoms of the disease. No abnormalities in his full blood count and biochemistry. Pediatric neurologist consult him—no symptoms of meningeal irritation, Bell’s symptom (+) on the left side, impossible contraction of m. frontalis sin., m. orbicularis oculi sin. and m. orbicularis oris sin., without other pathological abnormalities for the rest of cranial nerves. Muscle tonus—normal. Tendon reflexes—symmetrical and without deviations. Treatment for the peripheral Bell`s palsy was initiated.

**Figure 2:**
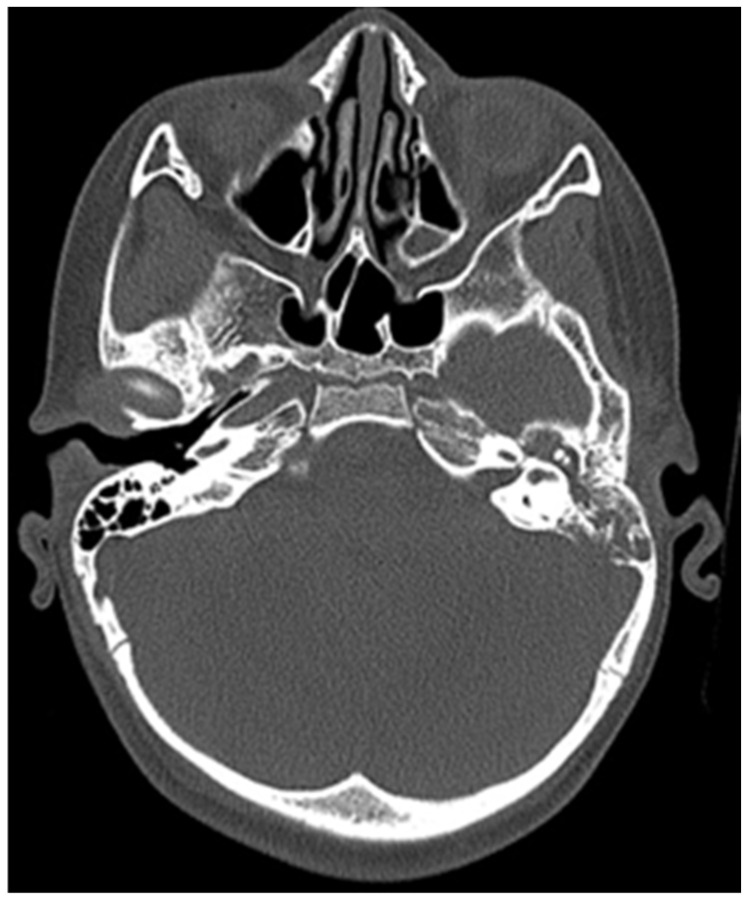
Initial CT of the patient.

**Figure 3:**
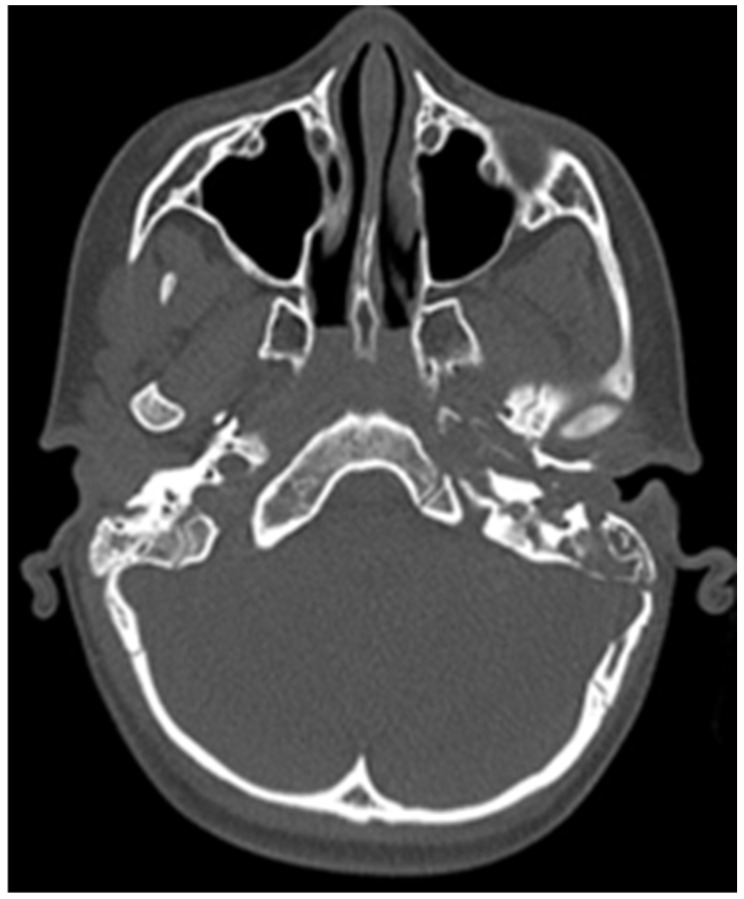
Initial CT of the patient.

CT of the head was performed, which showed destruction of the left mastoid process from a soft tissue tumour mass, which involves the cavities of the middle ear, tympanic cavity and the external auditory channel (Figure 2 and Figure 3).

After these CT findings, an examination by an ENT specialist was performed—left external auditory canal was completely obstructed by a soft tissue formation, which resembles granulation tissue, left tympanic membrane was impossible for observation. Second diagnosis was established—peripheral facial nerve palsy, caused by middle ear chronic inflammation. So the patient was transferred to an ENT clinic for surgical treatment.

**Figure 4:**
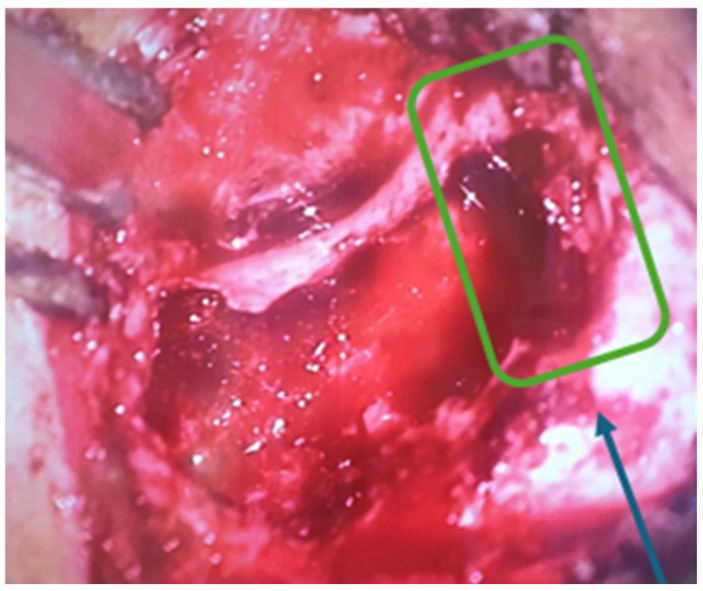
Exposed dura mater.

**Figure 5:**
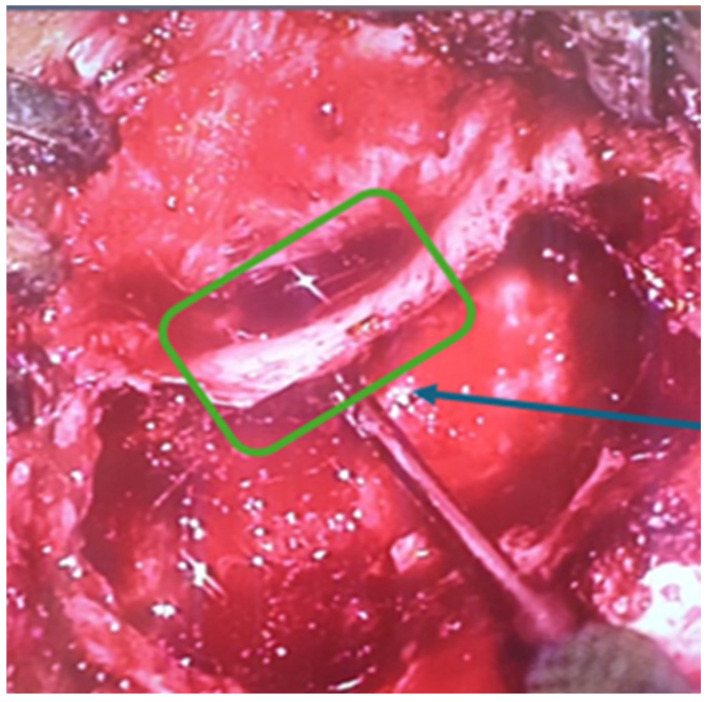
Dehiscence of the external auditory canal wall.

A surgical intervention was performed—radical trepanation. It started with retroauricular incision and mastoid bone exposure. As we started drilling of the mastoid bone, there was a destruction of the normal mastoid bone structure by a soft tissue mass, which was filling the entire mastoid beneath the cortex. The aditus was also blocked by that soft tissue. Following the destruction in a cranial direction, there was a 3 cm zone of exposed durra matter (Figure 4). After removing of the posterior wall of the external auditory channel, there was a large defect (Figure 5), through which the soft tissue was spreading from the mastoid to the external auditory channel. Step by step a classical radical trepanation cavity was formed. Tympanic membrane and ossicular chain remnants were visualized and removed. During the inspection of the tympanic cavity, a small tumour mass, which causes profuse bleeding if touched, was found in the area of the Eustachian tube hole. Intraoperative frozen section biopsies of the other soft tissue masses were all negative for malignant tumour cells and because of that, a decision for postoperative MRI around 30 days after the surgery was taken to clarify the surgical finding.

No postoperative complications were registered. Peripheral facial nerve palsy underwent total resolution in less than 24 h. The surgical wound healed without complications.

Histology:

1. External auditory canal—polypoid changes.
2. Mastoid cavity—fragments of granulation tissue.
3. Tympanic cavity—soft tissues with lymphoplasmatic infiltration
4. Immunology—negative for malignant processes

The child was discharged in excellent general condition with scheduled follow-up examinations. Fifteen days later, the patient came back for a follow-up exam with complaints of profuse purulent secretion from the left ear, relapse of the left sided peripherial facial nerve palsy, cough, general fatigue.

The examination showed an exophytic formation, bleeding profusely on palpation, which occupies the entire surgical cavity, left sided peripheral facial nerve palsy, convergent strabismus on the left side. Soft tissue formation presence in the area of the left Eustachian tube pharyngeal hole. A slight medial deviation of the left palatine tonsil was observed. Enlarged neck lymph nodes bilaterally with left side predominance were found.

**Figure 6:**
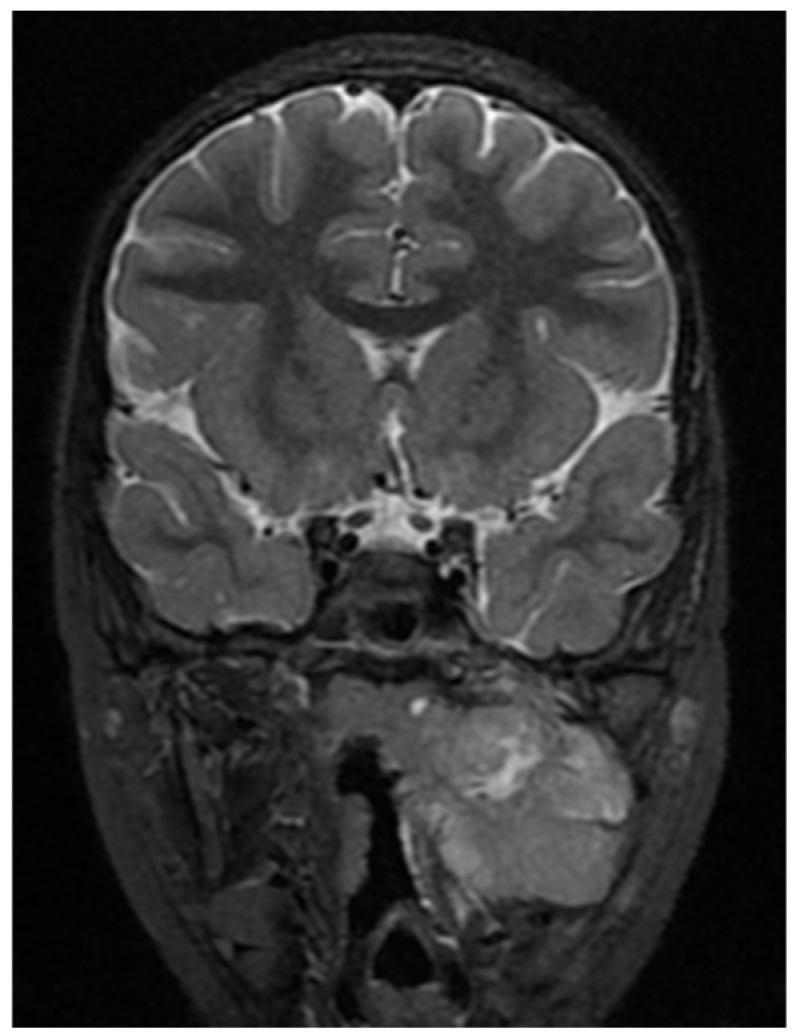
MRI of the patient on postoperative day 17.

**Figure 7:**
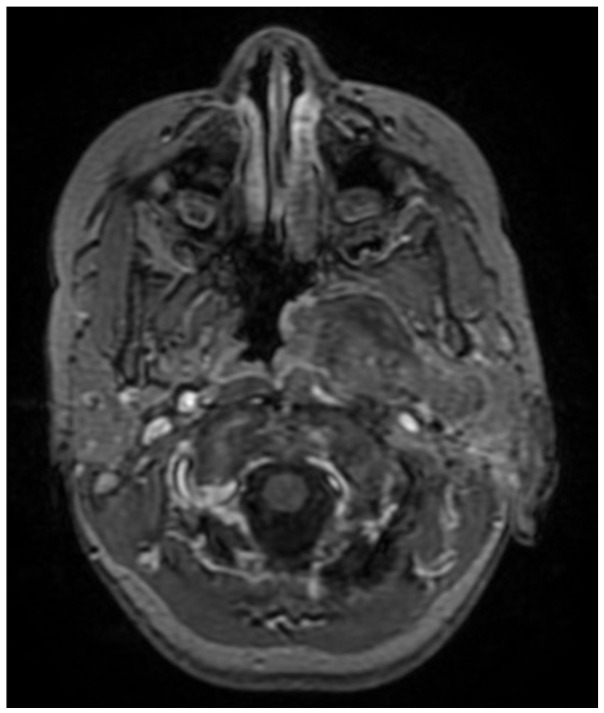
MRI of the patient on postoperative day 17.

**Figure 8:**
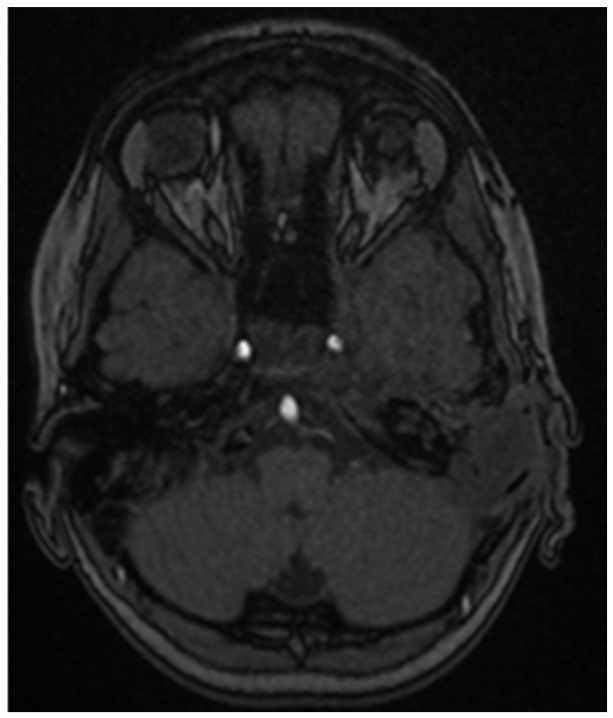
MRI of the patient on postoperative day 17.

A CNS MRI was performed, which reveal a large lobulated formation in the oropharynx and nasopharynx with left tonsil involvement, spreading to the retropharyngeal space and propagating through the left Eustachian tube into the middle ear. The previously formed surgical cavity was completely infiltrated from the soft tissue tumour mass. Approximate dimensions of the lesion in the mastoid—25/45 mm. axial, 38/39 mm. coronal (Figure 6, Figure 7 and Figure 8).

A biopsy of the tumor mass was performed from several places—surgical cavity, epipharynx and mesopharynx (left tonsil area), with frozen sections result—purulent inflammation and necrosis, the presence of atypical cells suspected. Morphology- polypoid tissue, covered by squamous epithelium. Under it, there is a dense infiltration of a tumor of so-called small blue round cell group. The tumor cells are with round to ovoid, hyperchromatic nuclei with only scanty cytoplasm. There are many mitotic and apoptotic figures. Immunohistochemistry-positivity for Desmin and EGF receptor. Furthermore, there is a positivity for Myogenin in a small subset of tumor cells. The staidly pattern for MyoD1. FISH showed negativity for PAX3/7 FOXO1. Conclusion—Embryonal Rhabdomyosarcoma.

Staging CT scan of the lungs and abdominal organs was performed- two right sided (6th and 10th segments, 8 mm and 5 mm, size respectively) and one left sided (1–2 segment, 4 mm size) pulmonary metastases were registered.

The initial PET CT at the diagnose do not reveal distant lymph node metastases, nor solid organ changes, no evidence for bone metastases and bone marrow activity. Stage 4, according to INRG-T_2_N_1_M_1_.

The child was transferred to the paediatric oncohematology department, where chemotherapy, according to CWS 2009 –CEVAIE risk arm was initiated. After the first six courses of chemotherapy, there was a total resolution of the peripheral left sided facial nerve palsy, a tumor size reduction in the area of the left ear without local bleeding, reduction of converging strabismus, total resolution of cervical lymphadenomegaly, as well as shrinking the tumor masses in the oropharynx and epipharynx

The restating PET CT revealed the persistence of the MRI soft tissue finding involving the oropharynx, nasopharynx, parapharyngeal, prevertebral and carotid spaces. Lysis of multiple bones was also visualized—temporal, clivus, pterygoid process of the sphenoid bone, mandible on the left as well as complete reversal of the previously described lung lesions.

**Figure 9:**
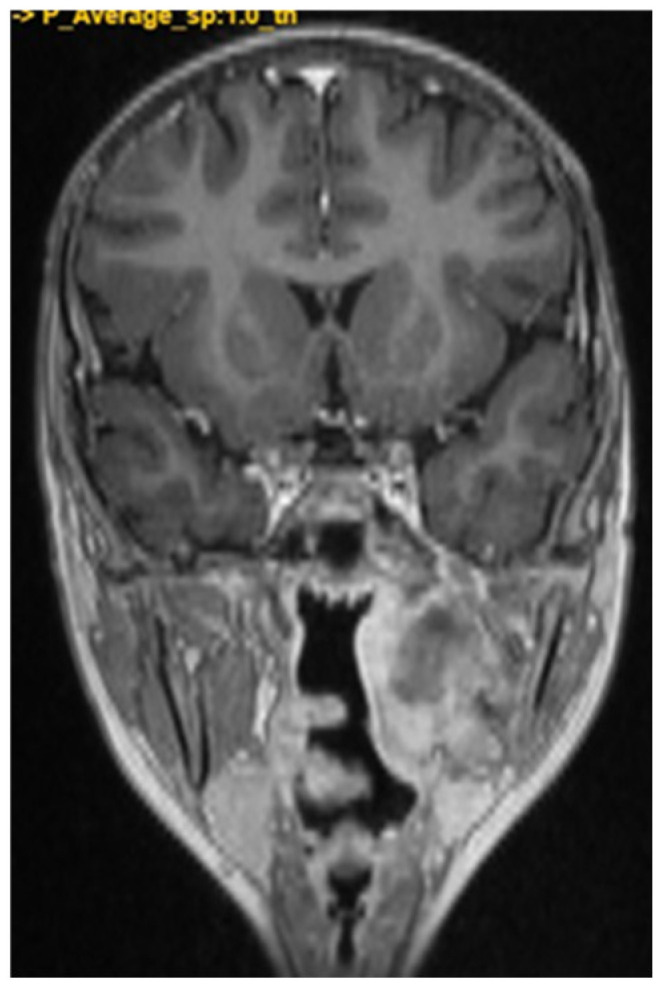
MRI on the 4th month from the start of chemotherapy.

**Figure 10:**
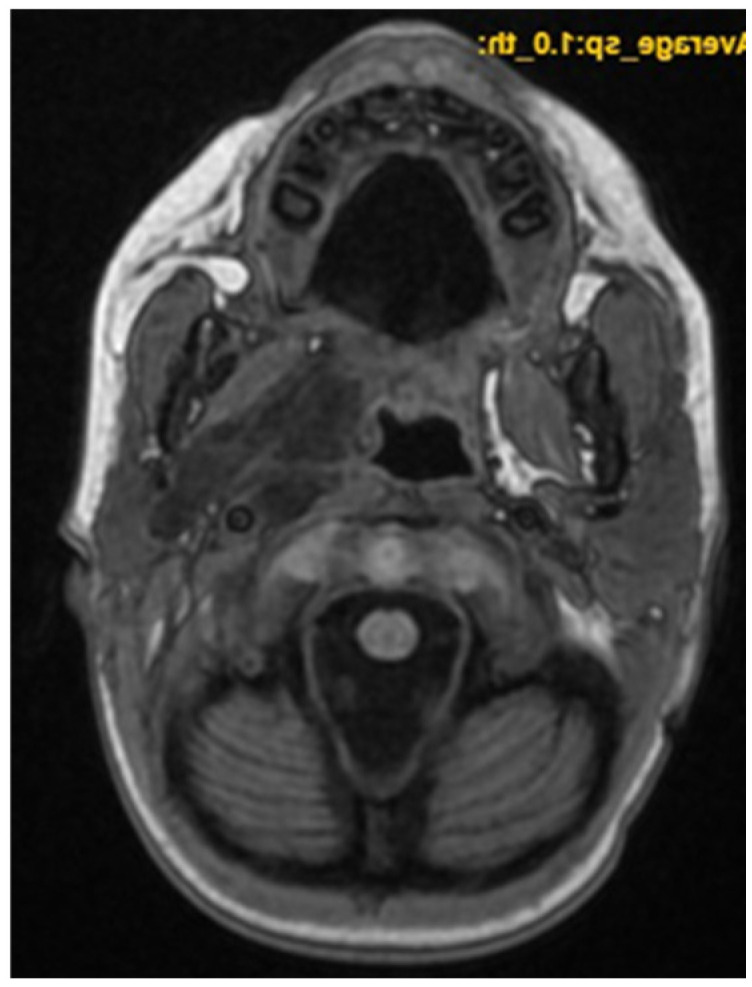
MRI on the 4th month from the start of chemotherapy.

**Figure 11:**
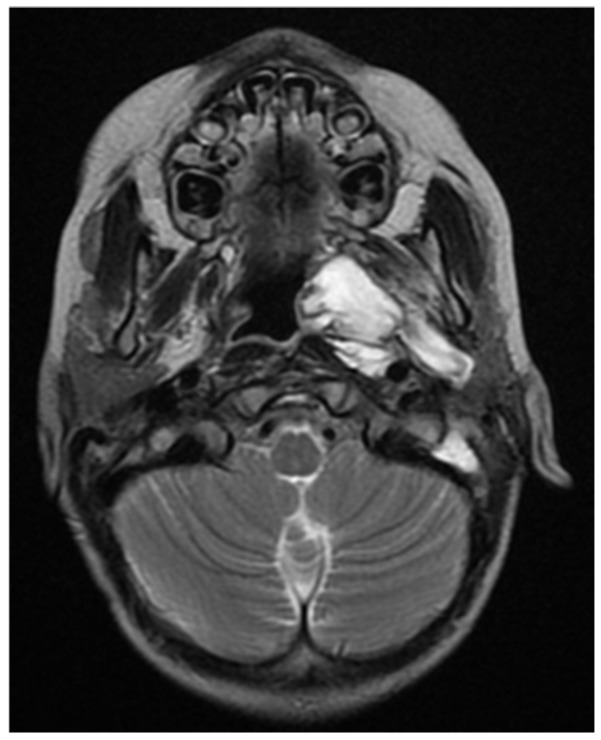
MRI on the 4th month from the start of chemotherapy.

The restating MRI before the fourth chemotherapeutic block showed persisting tumor mass in the nasopharynx and oropharynx with reduced size in the different dimensions 15/44 mm axial size and 31/40 mm coronal size. The lesion involves the vascular nerve bundle in the area of the cavernous sinus. In the skull base area, the formation was closely adjacent to the internal carotid artery. Full morphologic response to the cervical lymph nodes. (Figure 9, Figure 10 and Figure 11).

A. local irradiation (Electa lineal accelerator)in the zone of the primary tumour up to 36Gy was initiated after the restaging MRI. A new CNS and neck MRI is planned for December in order to evaluate the further shrinkage of the tumor and to plan the remaining dose of local irradiation of the residual disease.

For all procedures, the medical team received approval from the child’s parents and an informed consent was signed. No approval from an ethical-scientific committee was requested because the applied surgical treatment followed the nationally approved guidelines, and all other steps of the treatment were in accordance with internationally approved protocols.

## 3. Discussion

Middle ear Embryonal Rhabdomyosarcoma is a common solid tumor, which could mimic middle ear inflammation or mastoid inflammation in patients less than 10 years—most of the cases are between 2 and 5 years of age. These tumors have invasive growth and could destruct the middle ear, orbit, parapharyngeal space, the nasopharynx and can also extend its growth to fossa pterygopalatine.

In most cases surgical treatment strategy is to perform only a biopsy from the solid part of the tumor mass (mainly from the oropharynx or the mastoid if it is infiltrated by the tumor) to prevent false negative biopsy, which could lead to delay of the diagnose and treatment. Radical surgical tumor removal in these areas is usually impossible at the time of diagnosis but if achieved, full recovery can be expected. The differential diagnosis for such a localization is mainly with EXING sarcoma, so it is important to have pathohistological examination for MyoD1(which confirms RMS), CD 99 and EWSR1 gene evaluation (which could confirm EWING sarcoma).

According to the risk stratification of both SIOPE guidelines and the CWS 2009 protocol, these tumors are high risk or very high risk depending of the metastatic status of the disease and the Intergroup Rhabdomyosarcoma Study (IRS) group status, which is most frequently III. The evidence for lymphogenic spread of the disease is also very common.

The most common treatment modalities for this tumor as local control is the radiotherapy(photon, EBRT or proton therapy) and the systemic control is obliged to be mainly with multi-drug chemotherapy. Due to the poor prognosis of this patients, there is urgent need for novel therapies to be included especially for the pediatric patients.

## 4. Conclusions

Pediatric rhabdomyosarcoma of the head and neck is a rare pathology for the ENT surgeon. It has a rapid onset and in most cases presents with advanced disease. The majority of patients have cranial neuropathy at presentation [8].

Early signs such as facial pain, sinonasal congestion, and ear pain are relevant for many benign pathological conditions and thus can be overlooked for early tumor presentation [8,18].

Rhabdomyosarcoma, involving the parameningeal regions, including the nasopharynx, paranasal sinuses, and temporal bone, is usually not amenable to complete surgical resection. In these areas, radical surgery carries the risk of major cranial nerves damaging and secondary facial skeleton abnormalities.

The multimodal approach seemed to be the ideal management of RMS. It involves a combination of chemotherapy and local control with surgery and/or radiotherapy [3,19]. Surgery in these areas is usually limited to taking biopsy only.

Based on the clinical findings of that single case we cannot extract proper recommendations for future research and treatment, but a few suggestions can be made:

1. It is necessary to pay attention to minimal complaints suggesting that RMS may be the underlying cause of acute or chronic otitis media.
2. Imaging tests are essential and bone destruction caused by soft tissue mass must be under suspicion.
3. Surgery must be done to verify the process(biopsy) or in rare cases to eradicate the tumor masses.
4. The leading treatment must be chemotherapy.
5. Severe comorbidity can be expected during the chemotherapy courses.
6. The creation of a multidisciplinary team is of great importance for the treatment of the disease and dealing with the complications associated with it.

## Figures and Tables

**Table 1:** TNM pretreatment staging classification [8,13].

Stage	Site	T	Size	N	M
I	Orbit, head and neck (excluding parameningeal)	T_1_ or T_2_	a or b	N_0_ or N_1_ or Nx	M_0_
II	Parameningeal	T_1_ or T_2_	a	N_0_ or Nx	M_0_
III	Parameningeal	T_1_ or T_2_	a b	N_1_ N_0_ or N_1_ or Nx	M_0_ M_0_
IV	All	T_1_ or T_2_	a or b	N_0_ or N_1_ or Nx	M_1_

T_1_—tumor confined to anatomic site of origin, T_2_—tumor extension and/or fixative to surrounding tissue. Lesion size—a ≤ 5 cm in diameter and b > 5 cm in diameter. Lymph nodes involvement—N_0_, regional nodes not involved, N_1_, regional nodes involved and Nx, regional status unknown. Metastases—M_0_, no distant metastases or M_1_, metastases present (includes positive cytology in CSF).

**Table 2:** The Intergroup Rhabdomyosarcoma Study Clinical Grouping System.

Group	Definition
I	Localized disease, completely resected (A) Confined to muscle or organ of origin (B) Contiguous involvement, with infiltration outside the muscle or organ of origin; regional nodes not involved
II	Compromised or regional resection (A) Grossly resected disease with “microscopic residual” (B) Regional disease, completely resected, in which nodes may be involved and/or extend into an adjacent organ (C) Regional disease with involved nodes grossly resected, but with evidence of microscopic residual
II	Incomplete resection or biopsy, with gross residual disease
IV	Distant metastatic disease present at onset

## Data Availability

The data presented in this study are available on request from the corresponding author. The data are not publicly available due to ethical restrictions.
